# Moral competency of students at a german medical school – A longitudinal survey

**DOI:** 10.1186/s12909-024-05674-x

**Published:** 2024-06-26

**Authors:** Stephan Nadolny, Florian Bruns, Andre Nowak, Jan Schildmann

**Affiliations:** 1 Institute for History and Ethics of Medicine, Interdisciplinary Center for Health Sciences, Martin Luther University Halle-Wittenberg, 06112 Magdeburger Str. 8, Halle, Germany; 2 Institute for Educational and Health-Care Research in the Health Sector, Hochschule Bielefeld – University of Applied Sciences and Arts, Bielefeld, Germany; 3 Institute for the History of Medicine, Faculty of Medicine Carl Gustav Carus, TU Dresden, Dresden, Germany; 4 Clinical Ethics and Ethics Committee, University Hospital Schleswig-Holstein, Lübeck/Kiel, Germany

**Keywords:** Medical students, Ethical competence, Moral competence, Survey, Germany

## Abstract

**Background:**

Medical students and doctors face various challenges in clinical practice. Some of these challenges are related to ethical issues. Therefore, teaching ethics respectively building moral competences has become an integral part of the medical curriculum in Germany and many other countries. To date, there is little evidence on moral competence of medical students.

**Methods:**

Self-administered survey among medical students from one German medical school in the first (cohort 1) and fifth semester (cohort 2) in the winter term 2019/20 (T0). Both cohorts received the same questionnaire one year later in winter term 2020/21 (T1). Assessment was performed with Lind’s Moral Competence Test. We performed convenience sampling. We analyzed the data with descriptive statistics and C-Scores as a measure of moral competence (higher scores = higher competence, ≥ 30 points = high competence).

**Results:**

A total of 613 students participated in the study (response rate 67.5%, *n* = 288 with data on both time points). 69.6% of the participants were female, the mean age was 21.3 years. Mean C-Score for both cohorts for T0 (first and fifth semester) is 32.5 ± 18.0 and for T1 (third and seventh semester) is 30.4 ± 17.9. Overall, 6.6% (T0) and 6.7% (T1) of respondents showed some but very low moral competence. 3.3% (T0) and 3.0% (T1) showed no moral competence. Additionally, students without prior experience in the healthcare system scored 3.0 points higher.

**Conclusions:**

Improvement of assessment of moral competence as well effective interventions are particular needed for supporting those students which have been identified to demonstrate little moral competences.

**Supplementary Information:**

The online version contains supplementary material available at 10.1186/s12909-024-05674-x.

## Background

Medical students and doctors face a variety of ethical challenges in clinical practice. For particularly challenging situations, healthcare professionals in many institutions can rely on for example ethics committees [1]. However, healthcare professionals deal with moral problems in everyday practice which requires at least a certain degree of basic moral competence on the part of the individual to be able to act in a professional manner [2–4].

To be able to determine whether students or healthcare professionals possess moral competence clear definitions and criteria are needed. In the literature, moral competence has been conceptualized differently. The reasons for a rather heterogeneous understanding of moral competence are partly due to different understandings of “competences” or “expertise” and partly on (meta-) ethical differences with regard to tasks and scope of ethics [3, 5–8]. In their concept analysis Kulju et al. propose that ethical competence can be defined as a mixture of “…character strength, ethical awareness, moral judgement skills and willingness to do good” [7]. Lind uses a somewhat broader definition, stating that moral competence is “…the ability to solve conflicts and problems on the basis of moral principles through thinking and discussion instead of through violence, deceit or bowing down to others.” Next to this definition, Lind has developed an instrument to assess moral competence with an explicit theoretical foundation as well as thorough development [8].

In recent years in Germany as in many other countries ethics lectures and seminars have been introduced as part of the obligatory medical curriculum. However, there are considerable differences between amount and content of medical ethics training [9]. In addition, there is little evidence on the outcome of such teaching with regard to development of moral competence of medical students [10, 11]. Against this background, the aim of this study is to describe the level of medical student’s moral competences at one German university at different stages during the medical curriculum over the course of one year. Students in one cohort received ethics training (see Infobox 1 – Supplement 1) during the year which was subject of our survey. The findings of the study will be used as a starting point to explore potentials and limits of measuring moral competences as well as (interventional) research on moral or ethical competence in medical students.

## Methods

We conducted a longitudinal, self-administered, survey study.

### Population

The population was medical students in their first (cohort 1) and fifth semesters (cohort 2) at Martin Luther University Halle-Wittenberg (MLU) in Germany at the beginning of the lecture period in the winter term 2019/20. We have chosen these semesters to obtain basic data for the start of the course in medicine (cohort 1) as well as data before and after the History, Theory and Ethics module (HTE), which takes place in the fifth semester (cohort 2).

### Recruitment & sampling

Students were informed about the study with a short slide presentation by two student assistants in the last part of the first lectures of the 2019/20 term. The researchers were not present in order to avoid any form of conscious or unconscious pressure on the students to participate, as the researchers are also responsible for student assessment in the modules they teach. The student assistants then handed out the questionnaire, offered to answer questions and asked the students to evaluate their participation. They then left the room to give the students time for evaluation.

We employed convenience sampling, since we had no access to any personal data prior to survey administration.

### Data collection

Both cohorts received the same questionnaire in the winter term 2019/20 (T0) and one year later in winter term 2020/21 (T1). At T1 we were not able to use paper-based questionnaires due the COVID-19 pandemic and therefore changed the mode to an online survey.

The students got 15 min of time at the end of the first lectures to fill out the questionnaire if they opted to participate. They could also take the questionnaires with them for later completion.

The completed paper-based questionnaires used in winter term 2019/20 could be returned to prominently placed boxes at the medical faculty. In winter term 2020/21 we used Limesurvey Software hosted on the servers of the MLU for the online survey. In the first, second and fourth week after the invitation, we sent reminders via e-mail and the internal elearning system.

### Questionnaire

The questionnaire comprised of (1) a short letter of clarification about the study, (2) sociodemographic data such as age, sex and prior work experience in healthcare, (3) a self-generating code page to allow for longitudinal analyses in the following year, comprising of the first two letters of the mother’s first name, the birth month of the mother (if the mother’s was not known the father’s or grandmother’s data was used) and the number of siblings, and (4) Lind’s German version of the Moral Competence Test (MCT) [8].

The MCT, which was called Moral Judgement Test until 2014, aims to measure moral competence based on Kohlberg’s stages of moral development. It comprises of two dilemmas:

The first one is a worker’s example in which some workers were fired. Some remaining workers suspect their authorities to be observing them through cameras and microphones, which the company denies. Therefore, two workers break into the office and take tapes, which provide evidence of observation.

The second dilemma is a doctor’s example in which a woman suffering from cancer with severe pain and frailty asks her doctor, in a brief period of improvement, to give her a lethal dose of morphine. She states, that she could not endure any further pain and would die anyway. In the end, the doctor applied the lethal dose of medication.

For both dilemmas the survey respondents were asked whether they would agree with the decision on a seven-point scale ranging from − 3 (I strongly disagree) to + 3 (I strongly agree). Afterwards, twelve arguments (6 pro and 6 contra) for each dilemma were presented. Each argument corresponded with a stage of Kohlberg’s stages of moral development [12]. The participants rated each argument for acceptability on a nine-point scale ranging from − 4 (I strongly reject) and + 4 (I strongly accept).

The result of the MCT was measured with the C- Score which constitutes the participant’s ability to weigh arguments for and against a moral decision with regard to the argument’s moral quality [8]. The score ranges from 0 to 100 and can be categorized as (A) 0-4.9 corresponding to no moral competence, (B) 5-9.9 to some, but very low, (C) 10-19.9 to low, (D) 20-29.9 to sufficient, (E) 30–100 to high up to very high competence [13]. The calculation scheme for the C-Score can be found in the literature [14]. The MCT has been frequently used to measure moral competence over the past 40 years [8].

### Data analysis

We excluded questionnaires with missings in the MCT, since prevalence of questionnaires with missings was very low. In a first analytic step, we compared the intergroup differences between the two cohorts. In a second step, we compared the longitudinal changes within each group. We calculated C-Scores and compared the two study groups descriptively with cross tabulation and mean differences, since no random sampling was conducted and therefore inference statistics were not feasible [15]. The sociodemographic data were used to explain differences within and between groups. For the influence of age, we performed Pearson’s correlational analysis. We analyzed moral segmentation, which was defined as a difference in C-Scores for the worker’s and doctors example of at least eight points [16]. Data Analysis was performed with IBM SPSS Statistics Version 24.

## Results

A total of 613 students participated in the study, resulting in an overall response rate of 67.5%. 69.6% of the participants were female, the mean age was 21.34 years and 22.8% got prior professional experience in the healthcare system. A detailed differentiation of response rates and sociodemographic data per cohort and year is shown in Table 1.

**Table 1:** Sociodemographic characteristics

	T0	T1
Response rate, n (%)	315 (69.1)	298 (62.7)
Age, mean ± SD	21.3 ± 3.9	22.5 ± 3.9
Sex, n (%)		
Diverse	2 (0.7)	2 (0.7)
Female	211 (69.6)	209 (70.1)
Male	83 (29.7)	84 (28.5)
Prior professional experience in healthcare, n (%)	69 (25.1)	68 (23.1)

SD = Standard deviation

The mean overall C-Score for both cohorts, indicating the moral competence of respondents for T0 (first and fifth semester) is 32.5 ± 18.0 and for T1 (third and seventh semester) is 30.4 ± 17.9 which is equivalent to a high competence. For cohort 1 the scores are 34.7 ± 18.4 (T0 = first semester) and 33.4 ± 21.1 (T1 = third semester). For cohort 2 the scores are 29.8 ± 17.4 (T0 = fifth semester) and 27.8 ± 14.4 (T1 = seventh semester). Table 2 displays the C-Scores for all cohorts and time points. In addition, Table 2 presents data on “moral segmentation” which indicates a minimum of eight points difference for individuals between the two examples. This occurred for 77.9% of the students at T0 and 75.0% at T1. At T0 36.6% of students achieved at least eight points higher scores in the workers and 41.3% in the doctor’s example. At T1 32% in the workers example and 43.0% in the doctors who scored at least eight points higher (see Table 2).

**Table 2:** Competence scores (C-Scores) for ethical competence, rating of acceptance and rate of moral segmentation

		Overall		Cohort 1		Cohort 2	
		T0	T1	T0	T1	T0	T1
C-Score, mean ± SD		32.5 ± 18.0	30.4 ± 17.9	34.7 ± 18.4	33.4 ± 21.1	29.8 ± 17.4	27.8 ± 14.4
C-Score Worker, mean ± SD		46.8 ± 22.3	44.7 ± 21.4	47.9 ± 22.2	46.8 ± 21.8	44.6 ± 22.5	42.8 ± 19.9
C-Score Doctor, mean ± SD		49.6 ± 24.9	49.1 ± 22.5	52.7 ± 25.1	51.6 ± 23.2	45.3 ± 24.3	44.1 ± 25.1
Rating of acceptance, median, (Min-Max)							
Worker		-1 (-3–3)	-1 (-3–3)	-1 (-3–3)	-1 (-3–3)	-1 (-3–3)	-1 (-3–2)
Doctor		1 (-3–3)	2 (-3–3)	1 (-3–3)	2 (-3–3)	1 (-3–3)	2 (-3–3)
Moral Segmentation, n (%)		303 (77.8)	221 (75.0)	134 (76.1)	124 (73.8)	96 (80.0)	93 (78.2)

SD = standard deviation, Min-Max = Minimum to maximum values

Overall, 6.6% (T0) and 6.7% (T1) of respondents showed some but very low moral competence. 3.3% (T0) and 3.0% (T1) showed no moral competence according to Lind’s classification. High to very high moral competence was demonstrated overall at T0 by 51.5% and at T1 by 50.2% (see Table 3). The acceptance of the protagonists’ behavior in the two examples was elicited on a seven-point ordinal scale from − 3 (strongly disagree) to + 3 (strongly agree). On median both cohorts rated the worker’s behavior as a -1, indicating slight non-acceptance over both time points. The doctor’s behavior was rated as a + 1 at T0 and + 2 at T1 signaling a slight to medium acceptance.

**Table 3:** Categories of competence, n (%)

	Overall		Cohort 1		Cohort 2	
	T0	T1	T0	T1	T0	T1
No moral competence	10 (3.3)	9 (3.0)	5 (2.8)	4 (2.0)	5 (4.2)	5 (4.2)
Some, but very low	20 (6.6)	20 (6.7)	11 (6.3)	11 (6.4)	9 (5.8)	9 (6.0)
Low moral competence	51 (16.8)	51 (16.9)	24 (13.6)	24 (13.7)	27 (22.5)	27 (22.6)
Sufficient moral competence	66 (21.8)	71 (23.5)	37 (21.0)	41 (23.5)	28 (23.3)	34 (26.8)
High to very high moral competence	156 (51.5)	141 (50.2)	99 (56.3)	90 (53.2)	53 (44.2)	47 (40.4)

Arguments for and against the protagonists’ behaviors were rated on a nine-point ordinal scale from − 4 (strongly reject) to + 4 (strongly accept). The most accepted arguments for the doctor’s example (+ 2 points) were:

- “because the doctor had to act according to his conscience and what he believed was right. The woman’s pain made it right for the doctor to ignore his moral obligation to preserve life”.
- “because the doctor was the only one who could do what the woman asked; respect for her wish made him act the way he did”.

The most rejected arguments (-3 points) were:

- “because the doctor only did what the woman talked him into doing. He does not need to worry about negative consequences.“
- “because the woman would have died anyway and it didn’t take much effort for.
- “him to give her an overdose of a painkiller.“
- “because he could have had it much easier if he had waited and not interfered with the woman’s dying.“.

### Socio-demographic variables and moral competence

Regarding the influence of the sociodemographic variables on moral competence as measured with the C-Scores, there is a mean difference in favor of male students of 1.7 points. Students without prior experience in the healthcare system scored 3.0 points higher. Furthermore, students in first semester scored 4.8 points higher than students in the fifth semester as well as being of younger age was very weakly correlated with higher scores (*R* = 0.12).

## Discussion

This paper presents longitudinal data on moral competences of two cohorts from one German university covering the time from beginning of the first year till beginning of the second year of medical school (cohort 1) and the beginning of year three to beginning of year four (cohort 2). While we did not plan this study as an interventional study it is of interest for the analysis that the second cohort received the main part of the ethics training at the university in between T0 and T1.

Main findings are firstly a high to very high moral competence of half of the students in both cohorts. About 25% of students in both cohorts have low or even no moral competence. Secondly, students did not show improved moral competence scores one year after the ethics teaching module compared to scores prior to the teaching. Thirdly, there were differences with regard to age, female gender, increasing semester and professional healthcare experience with lower C-scores for moral competences. Additionally, with regard to the goal of improvement of ethical competence, we will discuss educational strategies and challenges.

Our results show higher overall levels of moral competences compared with previous studies on medical students from Australia [16], Brazil [17], the Czech Republic [18], Pakistan [19] and Portugal [20, 21], physicians from Chile [22], nursing students from the Czech Republic [23] and Portugal [24], midwifery students from Poland, and similar findings to medical students from Poland [25], Portugal [17] as well as Germany [11].

An interesting result was that there was a slight decrease in competence in cohort 2 for the students who had received teaching on ethics during the year. This finding may be interpreted in at least two different ways and used for future (interventional) studies. On the one hand the findings raise questions regarding the influence of HTE teaching as currently delivered. On the other hand, the findings may be used as a starting point to clarify the goals of HTE training and the scope of the moral competence test according to Lind. According to Lind the MCT measures the “*ability to rate arguments according to their moral quality”* [8]. This measurement seems much narrower than the requirements to solve complex ethical issues in clinical practice. In a more recent article Kühlmeyer et al. [26] argue that based on Tanner & Christen’s [27] concept of moral intelligence there are five dimensions of ethical competence:

1. “The ability to develop a moral compass (reference system).
2. The willingness and ability to prioritize and strive for moral goals.
3. The ability to recognize and identify moral issues.
4. The ability to develop and determine a morally satisfactory course of action.
5. The ability to build moral behaviors by acting consistently and courageously” [27].

While such nuanced conceptualization of moral or ethical competence seems much in line with the goals of current HTE training of many medical schools in Germany [9] there is a lack of instruments empirically robust as the MCT to measure respective competences.

Even considering the above-mentioned possible limitations of the MCT to demonstrate findings relevant for ethical competences in medical practice, it seems a particular worrisome finding that about 25% of students show low or no moral competence. This finding is stable over both time points. Given the repeated discussions about professional misconduct of physicians, one question is whether the MCT or other tests may be used as possible screening tests to identify particular training needs.

The decline in competence was not entirely surprising, however, given the available evidence, which we will discuss in relation to the influence of socio-demographic variables. In this respect, it must first be noted that all differences may be due to chance, since we could only use a convenience sample. Competence was lower in the fifth semester students than in the first semester students, which could indicate a regression of moral competence over the course of studying medicine. The decrease in competence in age and semester is similar to other studies [16–18, 23]. Common explanations for this are the low rates of ethics education, small-group discussions and overload of information over the course of the study as well as the late contact with real-life practice [10, 17, 28]. Other explanations might be an increase in cynical attitudes as well as a decrease in empathy especially when getting in contact with hands-on experience [23]. The gender difference rather seems by chance, since it is usually reported as small and not statistically significant in other studies [16–20, 22]. The difference between participants with previous work experience is obviously strongly correlated with age. However, the lower scores could be explained with the same hypotheses as the decline over time, since those students got even more time in the healthcare sector.

In summary, this study shows a decline of moral competences as measured with the MCT even for the period of time in which the majority of ethics teaching took place. While we perceive our topics taught in the seminars to reflect a wide variety it should be pointed out that in the future it will be necessary to align the teaching in light of the currently revised National Catalogue of Learning Objectives in Medicine (NKLM, www.nklm.de). In this context we hope that there will be more teaching units available for the implementation of ethical topics into the medical studies. Independent of this possible development it will be necessary to investigate appropriate interventions as well as measurements to be able to demonstrate possible effects of ethics teaching on moral competences of medical students.

### Limitations

A main limitation is the convenience sample, however, we managed to achieve a decent response rate of 67.5%, which is higher [20], comparable to [16] or lower [24] than in similar studies. We cannot reflect on other surveys with convenience samples, since they often do not report the response rate. Convenience sampling could introduce bias, as it is likely that students who were more open to the topic would have participated. This might be indicated by the overall high level of moral competence. Nevertheless, the sample contains a considerable diversity of scores, with about 25% of the students having lower moral competence.

We did not assess religion as a variable, which could have explained some variation especially with regard to moral segmentation.

## Conclusion

This study shows high scores for moral competence for the majority of researched medical students. However, there remain questions regarding adequate measurements for moral competence. Improvement of screening, assessment as well as interventions are particular needed for supporting those students which have been identified to demonstrate little ethical competences.

Based on the results, we plan to further measure ethical competence in medical students with different assessments longitudinally from the first to the last semester.

### Electronic supplementary material

Below is the link to the electronic supplementary material.

Supplementary Material 1

## Data Availability

The datasets generated and/or analyzed during the current study are not publicly available due commitments made to the students, but are available from the corresponding author on reasonable request.
