# Supplementary material for: Moral competency of students at a german medical school – A longitudinal survey
Source: BMC Med Educ. 2024 Jun 26;24:691. doi: 10.1186/s12909-024-05674-x (PMC11201357; doi:10.1186/s12909-024-05674-x)
Supplement: Supplementary file 1 — Supplementary Material 1 [file 12909_2024_5674_MOESM1_ESM.pdf]

Supplementary file 1

Infobox 1 – Structure of the History, Theory and Ethics module at MLU

History, Theory and Ethics of Medicine<br>The current structure of teaching ethics in medicine at MLU consists of an HTE module in the fifth semester, as well as separate individual seminars on geriatric and palliative care in the seventh semester and research ethics in the ninth semester.<br>The main theoretical part consists of seven lectures in HTE and seven small-group seminars (90 minutes each) with a strong emphasis on case discussions.This is similar to other German medical universities.[9] Teaching takes place in monoprofessional classes.<br>For the seminars, the study group is divided into groups of about 20 students. The lectures are given by a medical ethicist and a historian. The seminars are given by a variety of experts in the field of medical ethics: ethics, medicine, nursing, philosophy of education, health economics, law. The seminars cover topics such as discrimination in health care, organ transplantation, abortion, clinical ethics with a strong focus on uncertainty of consent, end-of-life care, care under the Nazi regime, care in divided Germany, and the history of medicine in the city of Halle.<br>The learning outcomes are assessed in a single-choice exam.
